# Recommendations on fit-for-purpose criteria to establish quality management for microphysiological systems and for monitoring their reproducibility

**DOI:** 10.1016/j.stemcr.2024.06.007

**Published:** 2024-07-02

**Authors:** David Pamies, Jason Ekert, Marie-Gabrielle Zurich, Olivier Frey, Sophie Werner, Monica Piergiovanni, Benjamin S. Freedman, Adrian Kee Keong Teo, Hendrik Erfurth, Darwin R. Reyes, Peter Loskill, Pelin Candarlioglu, Laura Suter-Dick, Shan Wang, Thomas Hartung, Sandra Coecke, Glyn N. Stacey, Beren Atac Wagegg, Eva-Maria Dehne, Francesca Pistollato, Marcel Leist

## Main text

(Stem Cell Reports *19*, 604–617; May 14, 2024)

In the original version of this manuscript, there were instances where supplemental tables were superposed, leading to readability issues. To address this, the authors have revised the supplemental information to ensure that all tables are displayed correctly and can be easily interpreted. The layout of the tables has been adjusted to prevent any overlap, ensuring clarity and readability. The authors apologize for any inconvenience this may have caused and appreciate everyone’s understanding. The updated supplemental information should now provide a clear and accurate representation of the data.

